# Combined effects of elevated CO_2_ concentration and *Wolbachia* on *Hylyphantes graminicola* (Araneae: Linyphiidae)

**DOI:** 10.1002/ece3.5276

**Published:** 2019-05-29

**Authors:** Qichen Su, Xia Wang, Naila Ilyas, Fan Zhang, Yueli Yun, Chen Jian, Yu Peng

**Affiliations:** ^1^ The State Key Laboratory of Biocatalysis and Enzyme Engineering of China, College of Life Sciences Hubei University Wuhan China

**Keywords:** CO_2_ level, endosymbionts, *Hylyphantes graminicola*, nutrition, peroxidase, survival rate

## Abstract

The increasing concentration of carbon dioxide in atmosphere is not only a major cause of global warming, but it also adversely affects the ecological diversity of invertebrates. This study was conducted to evaluate the effect of elevated CO_2_ concentration (ambient, 400 ppm and high, 800 ppm) and *Wolbachia* (*Wolbachia*‐infected, W^+^ and *Wolbachia*‐uninfected, W^−^) on *Hylyphantes graminicola*. The total survival rate, developmental duration, carapace width and length, body weight, sex ratio, net reproductive rate, nutrition content, and enzyme activity in *H. graminicola* were examined under four treatments: W^−^ 400 ppm, W^−^ 800 ppm, W^+^ 400 ppm, and W^+^ 800 ppm. Results showed that *Wolbachia*‐infected spiders had significantly decreased the total developmental duration. Different instars showed variations up to some extent, but no obvious effect was found under elevated CO_2_ concentration. Total survival rate, sex ratio, and net reproductive rate were not affected by elevated CO_2_ concentration or *Wolbachia* infection. The carapace width of *Wolbachia*‐uninfected spiders decreased significantly under elevated CO_2_ concentration, while the width, length and weight were not significantly affected in *Wolbachia*‐infected spiders reared at ambient CO_2_ concentration. The levels of protein, specific activities of peroxidase, and amylase were significantly increased under elevated CO_2_ concentration or *Wolbachia*‐infected spiders, while the total amino content was only increased in *Wolbachia*‐infected spiders. Thus, our current finding suggested that elevated CO_2_ concentration and *Wolbachia* enhance nutrient contents and enzyme activity of *H. graminicola* and decrease development duration hence explore the interactive effects of factors which were responsible for reproduction regulation, but it also gives a theoretical direction for spider's protection in such a dynamic environment. Increased activities of enzymes and nutrients caused by *Wolbachia* infection aids for better survival of *H. graminicola* under stress.

## 1 INTRODUCTION

Because of the extensive use of fossil fuels since the industrial revolution, the atmospheric CO_2_ concentration has surged greatly from 280 ppm in the year 1700 to 400 ppm in the year 2015 (WMO, 2016). Elevated CO_2_ concentrations have raised the global average temperature, which directly affected the nutrition and biomass of the plants and insects, and even affected the agro‐ecosystems (Andresen et al., 2018; Lu et al., 2018). Although, previous studies have reported the effects of elevated CO_2_ concentration on the reproductive parameters such as development, fecundity, and oviposition rate in *Tetranychus urticae* (Acari: Tetranychidae), *Aphis gossypii* (Homoptera: Aphididae), and* Helicoverpa armigera* (Lepidoptera: Noctuidae) (Gao et al., 2008; Joutei, Roy, Impe, & Lebrun, 2000). However, these studies were mainly focused on the plants and insects, with little attention being focused on spiders.

Some studies have reported that elevated CO_2_ concentration can have potential effects on spiders. Zuo et al. (2015) reported that the total developmental duration of spiderlings was significantly increased at elevated CO_2_ concentration, while the body length and weight were decreased in a wandering spider, *Pardosa astrigera* (Araneae: Lycosidae). Unlike *P. astrigera*, when a web‐weaving spider *Agelena labyrinthica* (Araneae: Agelenidae) was exposed to elevated CO_2_ concentration, the total developmental duration was significantly decreased, while the carapace size and weight were not affected. These observations suggest that elevated CO_2_ concentration has species‐specific impacts on spiders (Wang et al., 2016).

Spiders can prey extensively on plant pests, decreasing the density of insects and stabilizing the populations, thereby effectively reducing the pesticide consumption (Maloney, Drummond, & Alford, 2003; Tanaka, Endo, & Kazano, 2000). In addition, spiders can be used as biological indicators to detect the heavy metals for monitoring environmental conditions (Li et al., 2016). They also have other advantages over other similar organisms. For example, the characteristics of greater resistance to temperature (low or high) and their higher resilience to starvation are uncommon in other predators (Cramer & Maywright, 2008; Henschel, Ward, & Lubin, 1992; Lowrie, 1980). *Hylyphantes graminicola* is the dominant species of the agricultural fields in China. They have a small body size (2.5–5 mm), a relatively fast growth rate, easily reared, and can prey on an extensive range of pests, like aphids, leafhoppers, and corn borers (Zhao, 1993). These characteristics indicate that *H. graminicola* is an ideal species for research in this field. As a consequence, *H. graminicola* also plays an important role in controlling pests in the agro‐ecosystems.

*Wolbachia* are maternally inherited Gram‐negative bacteria found in the reproductive tissues of arthropods. They belong to α‐subdivision of the *Proteobacteria* (Weisburg et al., 1989), and *Wolbachia* have attracted a considerable recent interest (Schuler et al., 2018). Interestingly, Duron et al. (2008) reported that the proportion of arthropod species have been infected from 22.8% to 32.4% in Western Europe, and such proportion had further increased to 40% (Zug & Hammerstein, 2012). *Wolbachia* are extensively widespread bacteria and their population has increased dramatically in nature. Although *Wolbachia* had a widespread effect in arthropods (LePage et al., 2017), few studies had reported the presence of these bacteria in spiders. Previous studies on *Wolbachia* have investigated their role in evolutionary processes (Yun, Lei, et al., 2011), reproduction and development (Yun, Yang, Wang, Peng, & Jiao, 2013), biological control (Kehlmaier, Michalko, & Korenko, 2012; Nikolouli et al., 2017), and transmission (Yun, Peng, Liu, & Lei, 2011). For example, a study conducted on spiders infected with *Wolbachia* showed that the female ratio of the spiders was increased (which is beneficial for distribution) (Goodacre, Martin, Thomas, & Hewitt, 2006), and no significant difference was shown in the reproduction in *H. graminicola* (Yun et al., 2013).

Oxidative stress is a common consequence of the metabolic disturbance in animals and thus can be explained to the responses to elevated CO_2_ (Matoo, Ivanina, Ullstad, Beniash, & Sokolova, 2013) and *Wolbachia* infection (Wong, Brownlie, & Johnson, 2015). Former studies indicated that elevated CO_2_ increased the pCO_2_/pO_2_ ratio (Pérez‐López et al., 2009), which induced an oxidative stress response on invertebrates (Tomanek, Zuzow, Ivanina, Beniash, & Sokolova, 2011). Excess reactive oxygen species (ROS) has been generated by *Wolbachia*, resulting in the production of the oxidative stress (Brennan, Keddie, Braig, & Harris, 2008), which affect the lipids, nucleic acids, proteins, and antioxidants of the host (Fridell, Sánchez‐Blanco, Silvia, & Helfand, 2005). This indicated that disturbances of oxidative stress balance may be a cocontribution of elevated CO_2_ and *Wolbachia*; however, the interactive effects of these two factors are not totally illustrated in spiders, which need further investigation.

A large number of former studies have reported a single effect of either elevated CO_2_ concentration or *Wolbachia* infection on invertebrate, but their interactive effect has been rarely studied. In addition, the ecological consequences of these interactions were not equal due to overlapping effects of the two factors (Murray, Ellsworth, Tissue, & Riegler, 2013). Former studies have proved that elevated CO_2_ levels or *Wolbachia* infection separately has significant effects on spiders (Goodacre et al., 2006; Wang et al., 2016; Zuo et al., 2015). With increasing of CO_2_ level and extending proportion of *Wolbachia* infection, *H. graminicola* would be certainly subjected to double stresses. At the same time, in view of the fact that both elevated CO_2_ and *Wolbachia* infection provoke strong oxidative stress, it seems interesting and logical to hypothesis that there existed interaction between elevated CO_2_ and *Wolbachia*.

In the year 2015, atmosphere CO_2_ concentration was 400 ppm, and it would be expected to rise to 970 ppm by the end of 21st century (Collins & Bell, 2004). According to the CO_2_ levels (400 ppm, approximately concentration in the current and 800 ppm, double concentration) and *Wolbachia* status (*Wolbachia*‐infected, W^+^ and *Wolbachia*‐uninfected, W^−^), we divided *H. graminicola* into four treatments: W^−^ 400 ppm, W^−^ 800 ppm, W^+^ 400 ppm, and W^+^ 800 ppm. So, to elucidate the interactive effects of the two factors on *H. graminicola*, the total survival rate, the developmental duration, carapace width and length, body weight, sex ratio, net reproductive rate, nutrition content, and enzyme activities of *H. graminicola* were examined in this study.

## 2 MATERIALS AND METHODS

### 2.1 Spider collection and rearing

Fifty‐eight female spiders were collected from wild corn (*Zea mays*) field located in Henan Province (112°49′E, 34°8′N), China, during September 2017. Most female adult spiders had exhibited copulatory behavior before capture in field. Female spiders were placed in cylindrical glass tubes (2 cm diameter, 6 cm high) with a layer of sponge (1.5–2 cm thick) moistened with water at the bottom, and bottleneck was plugged with cotton. These mother spiders were always kept under ambient CO_2_ concentration in a chamber at 28°C, with 60%–70% relative humidity, and L14:D10 photoperiod (light on at 08:00 hr). Most spiders laid their first egg sac in a week after we brought them to the laboratory and hatched in another week. The spiders were fed with adult *Drosophila melanogaster* without *Wolbachia* infection and cultured under ambient CO_2_ concentration.

### 2.2 Establishment of infected and uninfected *Wolbachia* spiders

Female spiders were used to extract DNA for *Wolbachia* infection screening when 2–3 egg sacs were laid. If the mother spider and 2‐s instar spiderlings from each egg sac were W^+^, then her offspring were confirmed as W^+^. The same method was used to test W^−^ spiders. *Wsp* (*Wolbachia* surface protein) gene was used to detect the infection of *Wolbachia* in spiders (Rowley, Raven, & McGraw, 2004). Genomic DNA was individually extracted following the manufacturer's protocols (CWBio), and assays for *Wolbachia* sequences were performed by PCR amplification using specific primers: 81F (5′‐TGG TCC AAT AAG TGA TGA AGA ACA C‐3′) and 691R (5′‐AAA AAT TAA ACG CTA CTC CA‐3′) (Braig, Zhou, Dobson, & O'Neill, 1998). The reaction system for *Wolbachia* used a temperature profile of 94°C for 30 s for initial denaturation, then 35 cycles of 94°C for 1 min, 56°C for 1 min, 72°C for 1 min, and a final extension of 72°C for 2 min. In 58 mother individuals, 25 spiders showed a *Wolbachia* infection (W^+^ group) and 33 spiders had no a *Wolbachia* infection (W^−^ group). After egg sacs hatched, spiderlings were separated individually into cylindrical glass tubes (one glass tube for each spider) and perpetually deposited into their respective CO_2_ concentration chambers until they died (ambient or high). *Wolbachia* infection was widely distributed in arthropods, and bioinformatics approach was essential for sequencing of *Wolbachia* strains which showed that most of *Wolbachia* belongs to subgroup A or B in most of arthropods (Pascar & Chandler, 2018). *Wolbachia* strain found in *H. graminicola* belongs to the supergroup B (Yun, Lei, et al., 2011; Yun, Peng, et al., 2011). Four treatments of *Wolbachia* and spiderlings under different levels of CO_2_ were used in the experiment. First treatment contained spiders without *Wolbachia* under 400 ppm of CO_2_ (W^−^ 400 ppm CO_2_). In 2nd treatment, spiders were reared under 800 ppm of CO_2_ without *Wolbachia* infection (W^−^800 ppm CO_2_). Spiderlings were infected with *Wolbachia* strain and reared under 400 ppm of CO_2_ concentration in 3rd treatment, while in 4th treatment *Wolbachia*‐infected spiders were reared under 800 ppm of CO_2_.

### 2.3 Assessment of total survival rate, developmental duration, length and width of the carapace, body weight, sex ratio, and net reproductive rate (*R*_0_)

All spiderlings (W^−^400 ppm treatment contains total 262 individuals, W^−^ 800 ppm of CO_2_ contains 257 individuals while W^+^ 400 ppm CO_2_ contains total 226 individuals and W^+^ 800 ppm of CO_2_ contains 220 ones) were selected randomly from 58 mother spiders. Survival rate is the ratio of number that spiderlings survive to maturity. Molts and reproduction were recorded during exuviation in the tube. The period of molts was used as a measure of developmental duration. The data of survival rate and developmental duration were observed at 6:00 p.m. every day until spiders were dead. They were identified to sexual organ after spiderlings were mature. The number of male and female was recorded to calculate the sex ratio. Three days postmaturation, 15 adult females *H. graminicola* were randomly selected from each treatment and weighed to the nearest 0.01 mg using an electronic balance (FA1004 N type, HANGPING). Then, these female spiders from each treatment were killed in 75% alcohol to measure the body lengths (from the front edge of carapace to the end of abdomen), using an ocular micrometer under a microscope (DFC495 type, LEICA).

Net reproduction rate of spiders (*R*_0_) is defined as the total number of mean number of descendants that a single spider can produce throughout its life time period and was calculated by the following formula: *R*_0_ = *N_t_*_+1_/*N_t_*. Where *N_t_* is egg number of beginning and *N_t_*_+1_ is egg number of next generations. We randomly selected 100 eggs (*N_t_*) from each treatment to rear, females that survive to maturity mated with males under the same treatment, and counted the total number of eggs laid by females (*N_t_*_+1_). Sex ratio is basically number of males and females in a population.

Sex ratio = Number of males/number of females × 100

### 2.4 Application of CO_2_ concentration

Two concentrations of CO_2_, ambient (400 ppm) (WMO, 2016) and high (800 ppm), were set up using CO_2_ artificial climate chambers (CC350TLHC type; Changzhou Okefenokee Instrument). The current ambient concentration of atmospheric CO_2_ is 400 ppm, so our “highest” treatment of CO_2_ was approximately double then the ambient level (Wang et al., 2016; Zuo et al., 2015).

### 2.5 Determination of nutrient composition and enzyme activity

Three days after the spiderlings molted, more than 25 individuals were randomly selected from each treatment and pooled into one sample, and replicated thrice. These individuals were used for the determination of nutrient composition and enzyme activity with the help of specific test kits. All kits were purchased from the Jiancheng Bioengineering Institute: quantitative total protein (A045‐2), total amino acid (A026), amylase activity (C016), and peroxidase activity (A084‐1). Bradford method was used for detection of proteins (Bradford, 1976). Absorbance of the sample was recorded at 595 nm wavelength (Deng et al., 2013). The shelf life of this kit was 6 months. Storage temperature was 2–8°C. This kit measured 0.2–1.3 mg/ml of sample, and it measured even four times more accurately then other methods. The POD activity is measure of change in absorbance at 420 nm (Li et al., 2013). The amylase activity was measured at 650 nm of wavelength according to instructions on kit. Kit examination range was 0–200 μmol/ml.

After weighing, spiders were added to 0.9% saline solution at 1:9 fresh weight‐to‐volume ratio. Optical density (OD) values were measured using an ultraviolet–visible spectrophotometer (UV‐6100, Shanghai Precision Instrument) and ELISA (SpectraMax Series Microplate Reader 190). Nutrients content and specific enzymes activities were calculated according to the kit instructions.

### 2.6 Statistical analysis

The developmental duration was analyzed by Mann–Whitney *U* test. Comparisons of sex ratio were done using chi‐square tests within variant CO_2_ concentration group or *Wolbachia* group. Comparisons of carapace width and length, body weight, net reproductive rate, nutrition content, and enzyme activity of *H. graminicola* were done using Student's *t* test within variant CO_2_ concentration group or *Wolbachia* group. The data were tested for normal distribution and homogeneity of variance using K‐S (Kolmogorov–Smirnov) test and Levene's test of equality of error variances, respectively. All interactive effects of CO_2_ concentration and *Wolbachia* infection were analyzed using two‐way ANOVA for significant comparisons.

## 3 RESULTS

### 3.1 Total survival rate of *H. graminicola*

**Figure 1:**
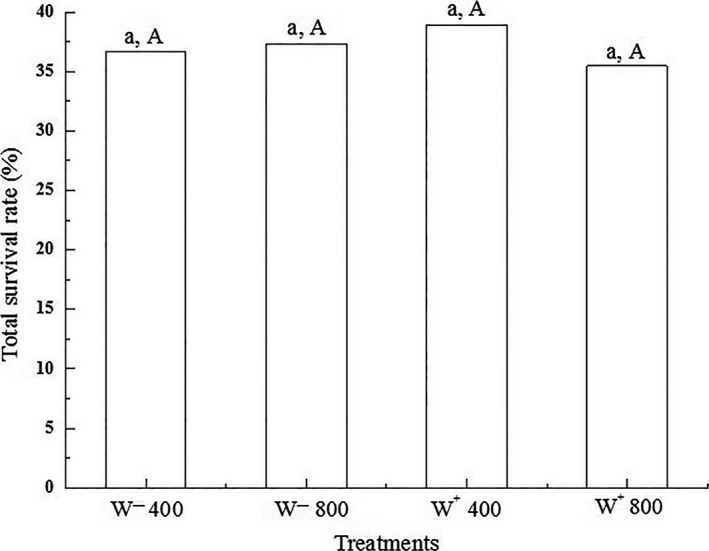
Survival rate in *Hylyphantes graminicola* with four treatments. Same lowercase letters show no significant difference between the two CO_2_ concentrations, and same uppercase letters indicate no significant difference between *Wolbachia*‐infected and *Wolbachia*‐uninfected (Student's *t* test, *p* > 0.05)

The total survival rate of *H. graminicola* in four treatments, W^−^ 400 ppm (*Wolbachia*‐uninfected with 400 ppm CO_2_), W^−^ 800 ppm (*Wolbachia*‐uninfected with 800 ppm CO_2_), W^+^ 400 ppm (*Wolbachia*‐infected with 400 ppm CO_2_), and W^+^ 800 ppm (*Wolbachia*‐infected with 800 ppm CO_2_), was 36.641% (96/262), 37.354% (96/257), 38.938% (88/226), and 35.454% (78/220), respectively. There was no significant difference using elevated CO_2_ concentration or *Wolbachia* infection (Student's *t* test, *p* = 0.693 and *p* = 0.942, respectively; Figure 1), and a significant synergistic effect of elevated CO_2_ concentration with *Wolbachia* infection was not found in *H. graminicola* (two‐way ANOVA, *p* = 0.893).

### 3.2 Developmental duration of *H. graminicola*

The total developmental duration of male *H. graminicola* was shorter than that of female spiders, and no significant effects were observed under high CO_2_ concentrations with *Wolbachia*‐uninfected spiders (Mann–Whitney *U* test, female: *p* = 0.402; male: *p* = 0.690). However, in the *Wolbachia*‐infected population of *H. graminicola*, a significantly shorter total developmental duration was observed (Mann–Whitney *U* test, female: *p* < 0.0001; male: *p* = 0.009). In addition, the developmental duration of second to fourth instar was significantly different between the two CO_2_ concentrations (*p* < 0.05; Table 1). These observations suggest that *Wolbachia* infection might be the main factor for total developmental duration and thus promoted the growth rate of *H. graminicola*.

**Table 1:** Mean (±*SD*) of developmental duration (days) in *Hylyphantes graminicola* instars with four treatments

Sex of spider	Instar	W^−^ 400	W^−^ 800	W^+^ 400	W^+^ 800
Female	Second	9.744 ± 1.177aA	9.209 ± 1.626aA	9.659 ± 0.938aB	9.086 ± 0.863bA
	Third	7.233 ± 1.770aA	7.581 ± 1.592aA	6.317 ± 1.128aB	7.014 ± 1.508bB
	Fourth	6.512 ± 2.028aA	6.860 ± 1.820aA	5.659 ± 1.621aB	5.629 ± 2.051bB
	Fifth	6.721 ± 1.790aA	6.884 ± 1.828aA	6.073 ± 2.078aA	5.857 ± 1.572aB
	Total duration	38.233 ± 4.173aA	37.372 ± 3.016aA	34.415 ± 4.171aB	33.700 ± 3.329aB
Male	Second	9.097 ± 1.165aA	9.333 ± 1.561aA	9.125 ± 0.906aA	8.844 ± 0.954aA
	Third	7.226 ± 1.146aA	7.100 ± 1.062aA	7.375 ± 0.941aA	7.094 ± 0.995aA
	Fourth	5.581 ± 1.911aA	6.433 ± 1.524aA	5.25 ± 2.184aA	5.594 ± 1.434aB
	Fifth	6.645 ± 1.561aA	6.567 ± 1.675aA	5.656 ± 1.598aB	6.156 ± 1.568aA
	Total duration	36.323 ± 3.824aA	35.7 ± 3.207aA	33.438 ± 3.387aB	33.688 ± 2.999aB

Note

Different lowercase letters indicate significant difference between CO_2_ treatments within *Wolbachia*‐infected and *Wolbachia*‐uninfected spider. Different uppercase letters indicate significant differences between *Wolbachia*‐infected and *Wolbachia*‐uninfected spider within CO_2_ concentration (Mann–Whitney *U* test, *p* < 0.05).

### 3.3 Carapace size and body weight of *H. graminicola*

Carapace width of *Wolbachia*‐uninfected spiders was significantly shorter when reared with elevated CO_2_ concentration (Student's *t* test, *t* = 5.321, *p* < 0.0001) while, the carapace width of *Wolbachia‐*infected spiders was significantly decreased under high CO_2_ concentration (Student's *t* test, *t* = 4.574, *p* = 0.001). Compared with ambient CO_2_ treatment, no obvious difference was observed in carapace length under high CO_2_ concentration; however, carapace length was significantly decreased in *Wolbachia‐*infected spiders (Student's *t* test, *t* = 2.949, *p* = 0.008; Table 2). The body weight observations of *H. graminicola* were nonsignificant among the four treatments (W^−^ 400, W^−^ 800, W^+^ 400, and W^+^ 800; Student's *t* test, *p* = 0.598). The interactions of carapace width and length were significant (two‐way ANOVA, *F* = 8.017, *p* = 0.007; *F* = 7.127, *p* = 0.011), resulting in reduced carapace size, whereas body weight was statistically not significant (two‐way ANOVA, *F* = 0.659, *p* = 0.423).

**Table 2:** Mean (±*SD*; *n* = 15) of carapace width (mm), carapace length (mm), and body weight (mg) in *Hylyphantes graminicola* with four treatments

Treatments	Carapace (mm)		Weight (mg)
	Width	Length	
W^−^ 400	0.949 ± 0.030aA	1.191 ± 0.067aA	4.630 ± 0.796aA
W^−^ 800	0.875 ± 0.041bA	1.144 ± 0.053aA	4.201 ± 0.343aA
W^+^ 400	0.896 ± 0.049aA	1.164 ± 0.067aA	4.228 ± 0.344aA
W^+^ 800	0.857 ± 0.051aB	1.106 ± 0.059aB	4.131 ± 0.345aA

Note

Different lowercase letters indicate significant difference between CO_2_ treatments within *Wolbachia*‐infected and *Wolbachia*‐uninfected spider. Different uppercase letters indicate significant differences between *Wolbachia*‐infected and *Wolbachia*‐uninfected spider within CO_2_ concentration (Student's *t* test, *p* < 0.05).

### 3.4 Sex ratio and net reproductive rate (*R*_0_) of *H. graminicola*

**Figure 2:**
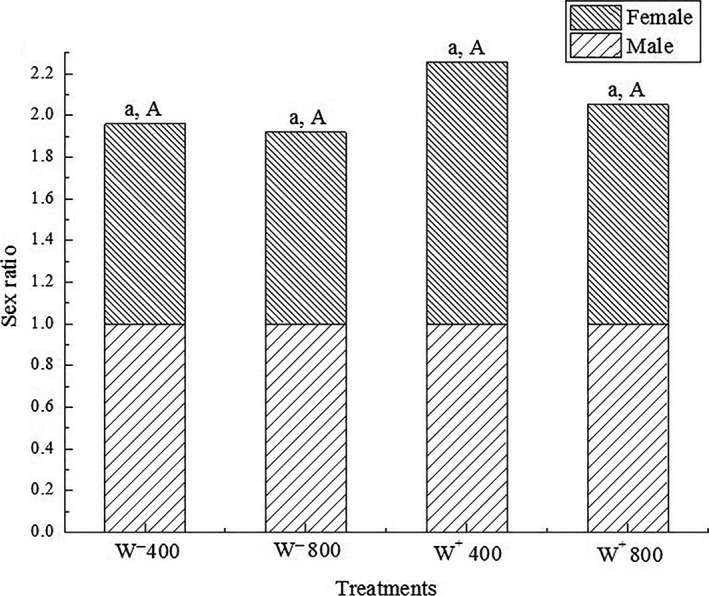
Sex ratio in* Hylyphantes graminicola* with four treatments. Same lowercase letters show no significant difference between the two CO_2_ concentrations, and same uppercase letters indicate no significant difference between *Wolbachia*‐infected and *Wolbachia*‐uninfected (chi‐square test, *p* > 0.05)

**Figure 3:**
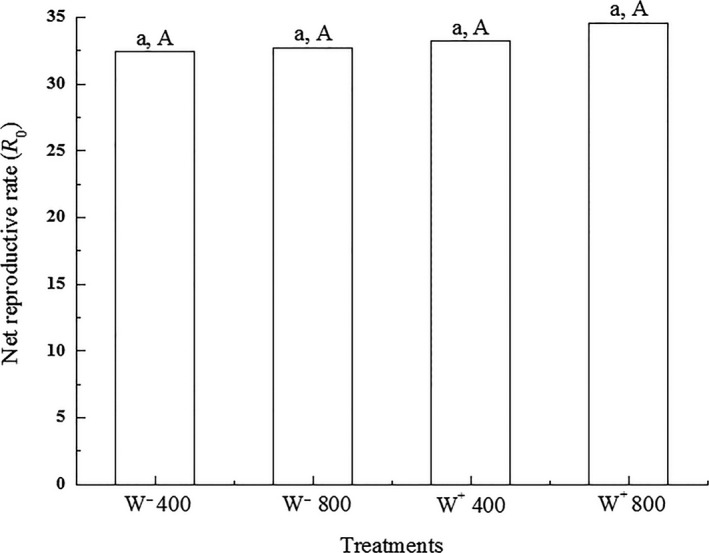
Net reproductive rate (*R*_0_) in* Hylyphantes graminicola* with four treatments. Same lowercase letters show no significant difference between the two CO_2_ concentrations, and same uppercase letters indicate no significant difference between *Wolbachia*‐infected and *Wolbachia*‐uninfected (Student's *t* test, *p* > 0.05)

The female:male ratios of W^−^ 400, W^−^ 800, W^+^ 400, and W^+^ 800 treatments were 0.959:1 (47/49), 0.920:1 (46/50), 1.256:1 (49/39), and 1.053:1 (49/38), respectively, which were not significantly different for either CO_2_ concentration or *Wolbachia* infection (chi‐square, *df* = 1, *χ*_0.05_^2^ = 3.84; *χ* ^2^ = 0.832, *p* = 0.363; *χ* ^2^ = 0.021, *p* = 0.363; *χ* ^2^ = 1.292, *p* = 0.256; and *χ* ^2^ = 0.007, *p* = 0.932, respectively; Figure 2). In addition, there was no significant difference on net reproductive rate of *H. graminicola* among the four treatments (W^−^ 400 = 32.471, W^−^ 800 = 32.732, W^+^ 400 = 33.284, W^+^ 800 = 34.562; Student's *t* test, *p* = 0.973), as shown in Figure 3. The interaction of sex ratio and net reproductive rate were also not significant in *H. graminicola* (two‐way ANOVA, *p* = 0.547 and *p* = 0.934, respectively).

### 3.5 Nutrient composition and enzyme activities of *H. graminicola*

Elevated CO_2_ concentration did not affect the amino acid content in *Wolbachia‐*uninfected spiders (Student's *t* test, *t* = 0.689, *p* = 0.496), but it was significantly increased in *Wolbachia‐*infected spiders (Student's *t* test, *t* = 5.904, *p* < 0.0001). Total protein content, peroxidase activity, and amylase activity were significantly increased under elevated CO_2_ concentration or with *Wolbachia‐*infected spiders (Student's *t* test, *p* < 0.05; Table 3). The interactions of *Wolbachia* and elevated CO_2_ concentration on total protein, amino acid content, peroxidase activity, and amylase activity were highly significant (two‐way ANOVA, *F* = 43.979, *p* < 0.0001; *F* = 34.696, *p* < 0.0001; *F* = 22.865, *p* < 0.0001; and *F* = 22.873, *p* < 0.0001), resulting in increased nutrient content and enzyme activity, which may be beneficial for *H. graminicola* in future.

**Table 3:** Mean (±*SD*; *n* = 3) of total protein, amino acid content, peroxidase activity, and amylase activity in *Hylyphantes graminicola* with four treatments

Parameter	W^−^ 400	W^−^ 800	W^+^ 400	W^+^ 800
Total protein (g/L)	0.293 ± 0.032aA	0.492 ± 0.021bA	0.208 ± 0.051aB	0.341 ± 0.124bB
Amino acid content (μmol/mg protein)	43.373 ± 10.465aA	46.085 ± 6.535aA	91.494 ± 32.581aB	142.109 ± 16.174bB
Peroxidase (U/mg protein)	1.477 ± 0.141aA	2.553 ± 0.502bA	4.454 ± 1.651aB	5.825 ± 1.405bB
Amylase (U/mg protein)	0.798 ± 0.681aA	0.808 ± 0.355bA	1.093 ± 0.056aB	2.010 ± 0.301bB

Note

Different lowercase letters indicate significant difference between CO_2_ treatments within *Wolbachia*‐infected and *Wolbachia*‐uninfected spider. Different uppercase letters indicate significant differences between *Wolbachia*‐infected and *Wolbachia*‐uninfected spider within CO_2_ concentration (Student's *t* test, *p* < 0.05).

## 4 DISCUSSION

*Wolbachia*, an endosymbiotic bacterium, widespread in insects and Arachnida, have important effects on growth, development, and reproduction. *Hylyphantes graminicola* is one of dominant species of spiders in the field of maize and cotton (Zhao, 1993). The effects of ecological environment caused by the gradually rising atmosphere CO_2_ concentration have attracted more and more attention. The survival rate of phytophagous insects varies with different species and host plant. Agrell, McDonald, and Lindroth (2000) reported that the survival rate of *Orgyia leucostigma* (Lepidoptera: Lymantriidae) was significantly declined under elevated CO_2_ concentration. In contrast, Osbrink, Trumble, and Wagner (1987) showed nonsignificant differences under different CO_2_ concentrations in *Trichoplusia ni* (Lepidoptera: Noctuidae). In this study, the total survival rate was not significant among the four treatments, and *H. graminicola* was not affected by the interaction of elevated CO_2_ concentration and *Wolbachia* infection. We speculated that only one generation of *H. graminicola* was reared and assessed under elevated CO_2_ concentration; therefore, the effects on total survival rate might be limited. Furthermore, a nonsignificant relationship was found between the total survival rate and *Wolbachia* infection in *H. graminicola*. Yun et al. (2013) and Zhang, Yang, Zhu, and Hong (2018) assessed *Wolbachia* as endosymbiotic bacteria that induce beneficial effect in spiders' growth and development process; this beneficial association was helpful in regulation of biological traits in spiders. Our findings coincide with them.

The response of elevated CO_2_ concentration in predatory insects is species‐specific (Ge, Chen, Wu, & Sun, 2010). The elevated CO_2_ concentration decreased the relative growth rate in cotton bollworm, *H. armigera* (Wu, Chen, & Ge, 2006), whereas the developmental duration was accelerated in *Sitobion avenae* (Homoptera: Aphididae) (Sun, Jing, & Ge, 2010). Similar results were found in different species in response to *Wolbachia* infection, whereby, different *Wolbachia* strains and host species exert different effects on fitness and growth. The developmental durations of prepupal stages in *Wolbachia* infection were significantly longer than uninfected *Trichogramma dendrolimi* (Hymenoptera: Trichogrammatidae) (Yang et al., 2008). In Mediterranean fruit fly (Diptera: tephritidae), *Wolbachia* infection shortened the total developmental duration, but it prolonged the embryonic development (Sarakatsanou, Diamantidis, Papanastasiou, Bourtzis, & Papadopoulos, 2011). Our study indicated that the total developmental duration was significantly decreased after *Wolbachia* infection, which proved that the *Wolbachia* infection accelerated the growth rate, and the total developmental duration did not significantly discriminate between the two CO_2_ concentrations in *Wolbachia* noninfected spiders. This phenomenon might be explained by the interaction of elevated CO_2_ and *Wolbachia* infection responses, but the definite molecular mechanism is still unclear.

Carapace width and weight are important tools for evaluating the development of spiders (Gonzaga & Vasconcellos‐Neto, 2002; Hagstrum, 1971; Jakob, Marshall, & Uetz, 1996). Our results showed that the carapace width was decreased significantly when exposed to high CO_2_ concentration, but carapace length and weight showed a nonsignificant effect. However, the effects on carapace size were similar to *P. astrigera* (Zuo et al., 2015). We speculated that *H. graminicola* can prey like a wandering spider (Zhao, 1993), so elevated CO_2_ concentration might affect respiratory rate (Miyashita, 1969), which results in decreased carapace width. In addition, individual effect of *Wolbachia* infection did not show a significant effect in carapace length, width, and weight, but interactions of the two factors (high CO_2_ concentration and *Wolbachia*) significantly shortened the carapace length and width, except body weight, suggesting that interaction of these treatments were major factors affecting the carapace size of the spiders, and carapace size would be an important parameter to be measured under any stress condition in future research.

Several studies have reported the sex ratio as a key parameter for determining the morphological effect on different spiders. The distortion caused by *Wolbachia* was obvious in *Anelosimus domingo* (Araneae: Theridiidae) and in *Stegodyphus dumicola* (Araneae: Eresidae) (Avilés & Maddison, 1991; Avilés, Varas, & Dyreson, 1999), while in solitary spiders, like *Pityohyphantes phrygianus* (Araneae: Linyphiidae), there was no direct relationship between *Wolbachia* infection and sex ratio variation (Gunnarsson, Goodacre, & Hewitt, 2009). Our data indicated that in *H. graminicola*, as a solitary spider, like *P. phrygianus*, the sex ratio was not affected by *Wolbachia*. Moreover, a relationship between sex ratio and elevated CO_2_ concentration has not been found. Accordingly, there was no significant interaction of elevated CO_2_ concentration and *Wolbachia* infection in *H. graminicola*.

The fertility of different insect species under elevated CO_2_ concentration varies (Chen, Wu, Ge, Parajulee, & Shrestha, 2005). Zuo et al. (2015) indicated that the total number of eggs was significantly decreased in *P. astrigera* under elevated CO_2_ concentration. However, the number of eggs laid by *H. armigera* did not differ between ambient and high CO_2_ concentrations (Chen et al., 2005). The same phenomenon was found with *Wolbachia* infection. For example, the number of eggs of *Wolbachia*‐infected *D. melanogaster* was increased significantly (Brownlie et al., 2009), while the number of eggs of *Triaeris stenaspis* (Araneae: Oonopidae) was not affected by *Wolbachia* infection (Korenko, Smerda, & Pekár, 2009). Our data illustrated that the net reproductive rate of *H. graminicola* was not significantly affected by elevated CO_2_ concentration or with *Wolbachia* infection, though it could be influenced by different *Wolbachia* strains and specific host species. Moreover, being exposed to elevated CO_2_ concentration for only a short time, it was hard to generate extremely significant effects. *Wolbachia* strain was beneficial in plant host of heterogonic gall wasp as it played a crucial role in the reproductive isolation of *Belonocnema treatae*. mtDNA diversity was present in two clades of western and eastern *B. treatae*, and symbiotic relationship was present between *Wolbachia* and gall wasp (Schuler et al., 2018).

Elevated CO_2_ concentration affect the nutrient composition and enzyme activity in *P. astrigera* and increase total protein content but peroxidase activity was not altered (Zhou et al., 2015). Unlike *P. astrigera*, the amylase activity in *A. labyrinthica* was higher and the peroxidase activity did not differ noticeably. These observations show that elevated CO_2_ concentration has a species‐specific impact on spiders. Molecular mechanism for the symbiotic relationship between *Wolbachia* and their hosts has not been clearly expressed, but the changes in enzymatic activity of insect hosts harboring *Wolbachia* have been reported (Henrichfreise et al., 2009; Melnikow et al., 2013). In our research, when spiders were infected with *Wolbachia* and reared under elevated CO_2_ concentration, the total protein, amylase activity, and peroxidase activity have been increased significantly. Amino acid content differed slightly but was not significantly increased under elevated CO_2_ concentration, though it clearly increased when using *Wolbachia*‐infected spiders. Total protein and amino acid content are indispensable for nutrition in insects, while amylase is an important hydrolase, and together they supply energy for survival and reproduction (Kong, Huang, Liu, & Liu, 2007; Sun, Jing, & Ge, 2009; Sun et al., 2010), thus these factors seem to be beneficial to *H. graminicola*. Peroxidase, an important protective enzyme in insects (Dubovskiy et al., 2008), catalyzes the decomposition of H_2_O_2_, which is required to maintain balance under conditions of oxidative stress (Ahmad & Pardini, 1990). Significantly, interactions of the two factors (elevated CO_2_ concentration and *Wolbachia* infection) on total protein, amino acid content, amylase activity, and peroxidase activity were found in *H. graminicola* and synergistically increased the nutrition content and enzyme activities.

In summary, in *H. graminicola*, an elevated CO_2_ concentration and *Wolbachia* infection were proved to be beneficial as it increases the protein contents and activity of some enzymes like peroxidase, amylase, and amino acids; thus, it aids in the development of spiders under dynamic environment but the impact on the reproduction of the spider was limited. Spiders are bioindicator of environmental pollution. Elevated level of CO_2_ is major threat to invertebrates existence and survival so rearing of spiders under stress condition (elevated CO_2_) with endosymbiotic bacteria *Wolbachia* might be helpful for understanding the complexity and adaptability of the *H. graminicola* population, and it will be used as biological control of other pests in the field so in this way ecofriendly strategy should be devised that will protect the environment from harmful effect of chemical pesticides and developmental duration of spider was accelerated. The elevated CO_2_ and *Wolbachia* infection enhance digestive enzymes and detoxification enzymes, it seems interesting and logical to hypothesis that there existed interaction between elevated CO_2_ and *Wolbachia*.

## CONFLICT OF INTEREST

All of the authors declare that they have no conflict of interest in the publication.

## AUTHOR CONTRIBUTIONS

Yu Peng and Jian Chen designed the experiments. Qichen Su, Xia Wang, Naila Ilyas, Fan Zhang, and Yu Peng conducted the experiments and data analysis. Qichen Su, Xia Wang, Yueli Yun, and Yu Peng wrote the manuscript.

## Data Availability

All essential data are available in the text.
